# 993. A CRISPR-Powered Universal Infectious Disease Assay

**DOI:** 10.1093/ofid/ofab466.1187

**Published:** 2021-12-04

**Authors:** Keith Brown

**Affiliations:** Jumpcode Genomics, San Diego, California

## Abstract

**Background:**

The COVID-19 pandemic has brought awareness to the dangers of emerging pathogens to global human health and welfare. Sensitivity and flexibility are important features for methods used to detect emerging pathogens. While PCR testing is rapid and sensitive, a significant advantage next generation sequencing (NGS) approaches have over PCR-based analyses is the ability to detect previously undiscovered pathogens while also providing genomic information that can detect SARS-CoV-2 genome sequence, identify source of co-infection, and the host transcriptional response in a single workflow. The critical component enabling this approach is Jumpcode CRISPRclean technology which removes abundant human and bacterial ribosomal RNA sequences from NGS libraries.

CRISPRclean workflow easily integrates into next generation sequencing projects

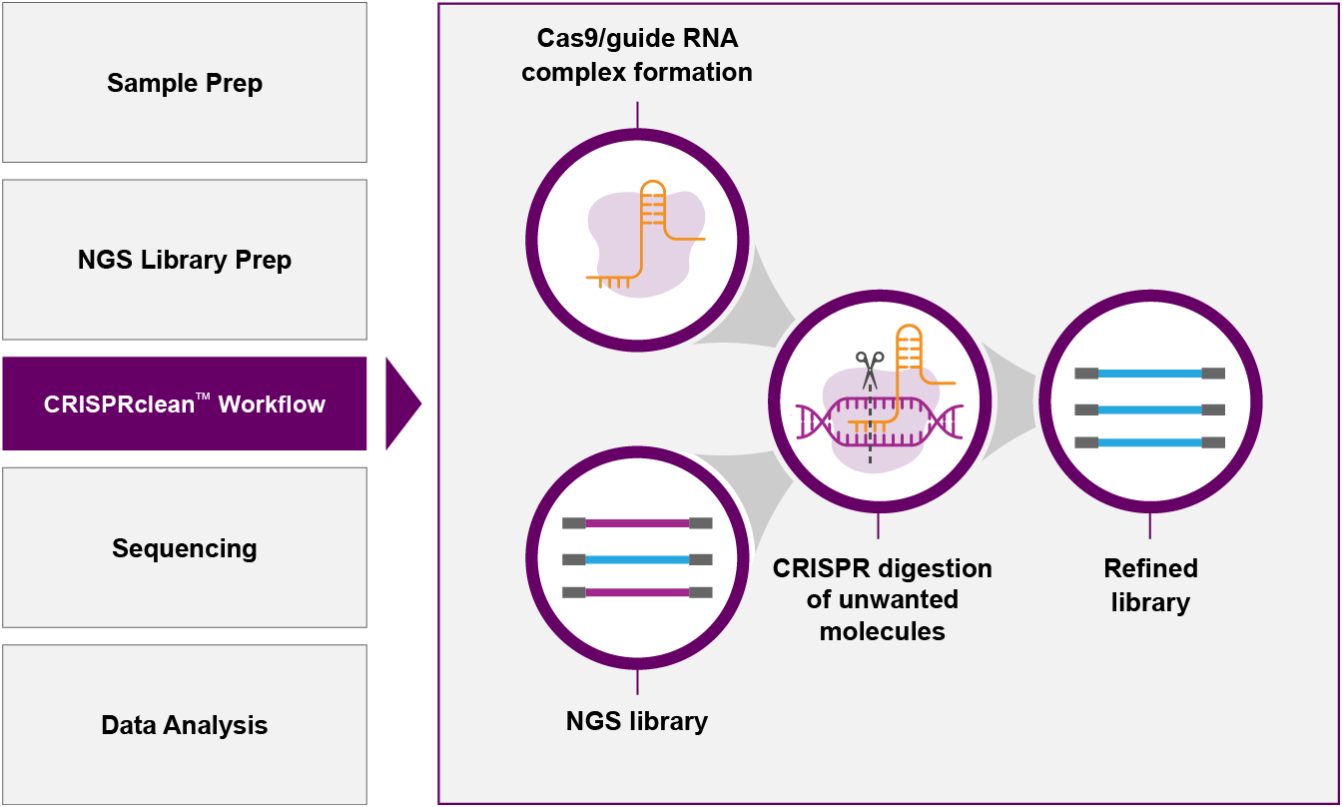

Schematic of the Jumpcode CRISPRclean protocol

**Methods:**

CRISPRclean was applied to contrived infected tissue samples including human lung RNA spiked with serially diluted amounts of SARS-CoV-2 RNA and bacterial RNA. NEB RNA libraries were prepared and treated with CRISPRclean protocol, then sequenced on Illumina instruments. Data analysis was performed using Jumpcode proprietary software to measure alignment and depletion rates, the Silva database for rRNA read alignment, and Kraken2 and CosmosID pipelines for k-mer based metagenomic investigation. Fold enrichment of SARS-CoV-2 reads after CRISPRclean depletion of libraries prepared from contrived samples.

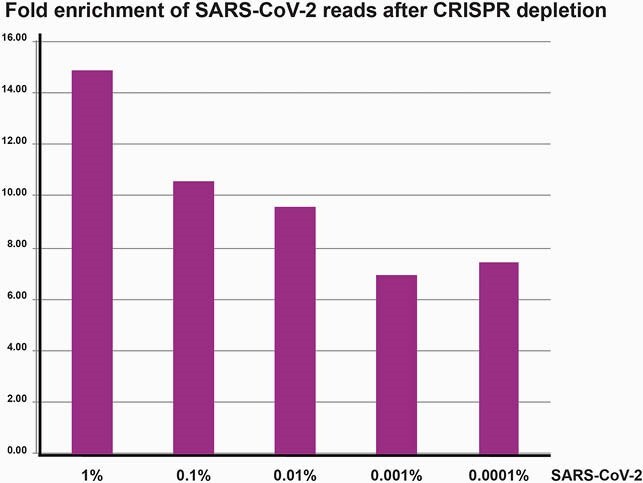

CRISPRclean treatment of the fully contrived samples increases the fraction of reads that map to the SARS-CoV-2 genome by an average of ~10-fold

**Results:**

CRISPRclean treatment of the contrived samples increases ~10 fold of reads that map to the SARS-CoV-2 genome. For the 60 viral copies of SARS-CoV-2 sample, the number of reads mapping to the SARSCoV-2 genome increases from ~10,000 reads to ~70,000 reads. A similar increase in reads occurs for S. aureus. The percentage of SARS-CoV-2 genome covered at 1X and 10X also increases. Similar results were achieved even after downsampling the datasets to 5M reads. There is a ~4-fold increase in bacterial species detection in these stool samples after CRISPRclean treatment. Percentage of SARS-CoV-2 genome covered at 1X and 10X increases as a result of rRNA depletion.

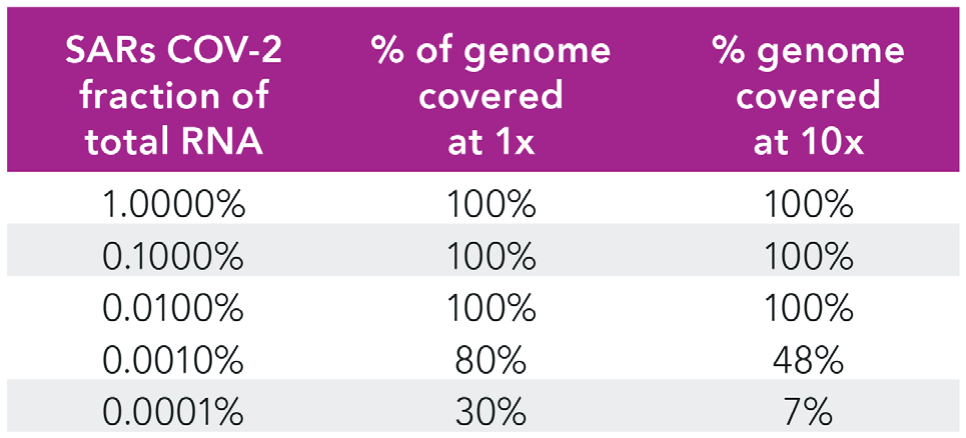

Coverage of the SARS-CoV-2 genome at 50 million reads.

Number of reads aligning to the S. aureus and SARS-CoV-2 genomes increases after CRISPRclean depletion.

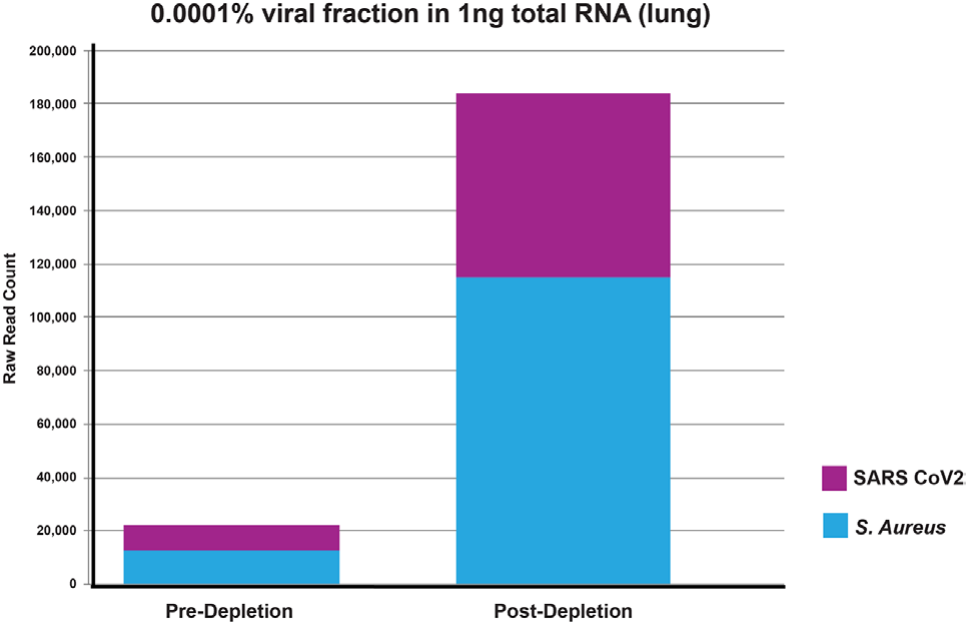

For the sample containing 0.0001% SARS-CoV-2, (60 viral copies), the number of reads mapping to the SARS-CoV-2 genome increases from ~10,000 reads to ~70,000 reads.

CosmosID Shotgun Metagenomics Analysis

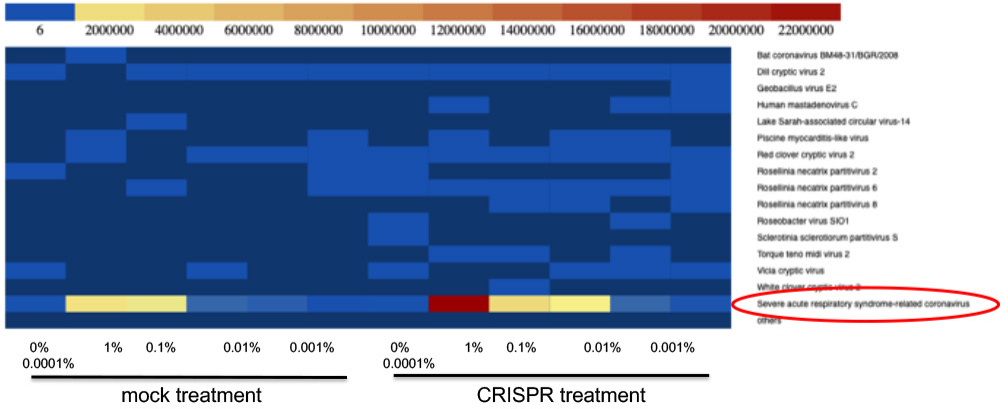

heat map showing read alignments to viral genomes. The yellow color indicates high read counts. The CosmosID shotgun metagenomic analysis software was used to analyze the sequencing data, classify the sequences and generate the heat map.

**Conclusion:**

Metatranscriptomics powered by CRISPR-mediated rRNA depletion offers a robust methodology to acquire viral genomic data, microbiome composition, co-infection information, and the transcriptional status of the host immune response in a single workflow. This sequencing-based approach can be available on the first day of the next viral outbreak and should be considered as a first-line test for novel zoonotic virus detection. Bacterial species composition of patient stool samples before and after CRISPRclean depletion.

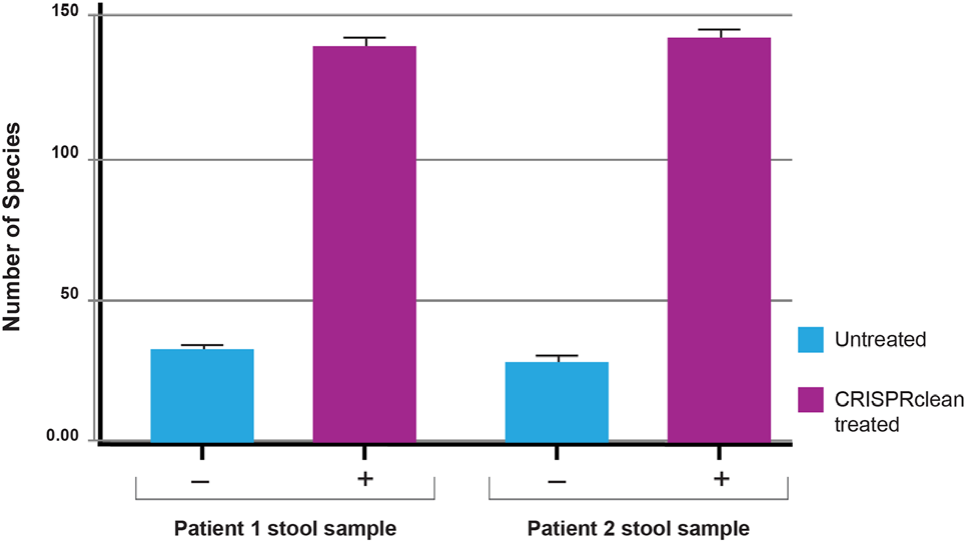

~4-fold increase in bacterial species detection in these stool samples after CRISPRclean treatment. Sequencing data downsampled to 20 million reads.

**Disclosures:**

**Keith Brown, n/a**, **Jumpcode Genomics** (Board Member, Employee, Shareholder)

